# Mood Disorders and BDNF Relationship with Alcohol Drinking Trajectories among PLWH Receiving Care

**DOI:** 10.4172/2329-6488.1000148

**Published:** 2014-02-10

**Authors:** María José Míguez-Burbano, Luis Espinoza, Mayra Vargas, Diana LaForest

**Affiliations:** 1 School of Integrated Science and Humanity, Florida International University, Miami, FL, USA; 2 Department of Medicine, University of Miami School of Medicine, Miami, FL, USA

**Keywords:** HIV, Hazardous alcohol, Alcohol trajectories, BDNF, Mood, Anxiety, Depression, Stress, Gender

## Abstract

**Background:**

Despite the excessive rates of Hazardous Alcohol Use (HAU) among people living with HIV (PLWH), although largely speculated, psychological and physiological components associated with HAU, has not been actively measured. Therefore, the present study was geared toward determining: 1) the rates of mood disorders and its relationship with HAU, and 2) to assess the impact of Brain Derived Neurotrophic Factor (BDNF), a well-known regulator of alcohol and mood disorders.

**Methods:**

For this study, participants of the longitudinal PADS Study n=400, were followed over time. Alcohol use (Alcohol Use Disorders Identification Test –AUDIT- and the Alcohol Dependence Scale –ADS) and moods (depression, anxiety, and stress) were assessed repeatedly.

**Results:**

A cluster analyses shows three distinctive trajectories. The first one, revealed a group with increased drinking (Cluster 1: n=140), constant alcohol intake (Cluster 2: n = 60), and one with decreased consumption (Cluster 3: n =120).

Analyses discovered higher AUDIT scores across the clusters with Cluster 1 being followed by Clusters 2 and 3 (1: 14.5 ± 8 vs. 2=8.7 ± 7.5 vs. 3= 6.6 ± 4.2, p = 0.001). Women in Clusters 1 and 2 had higher levels of stress (1:21 ± 7.5; 2:19.3 ± 7) and lower BDNF levels (7904 ± 1248 pg/ml and 10405 ± 909 pg/mL) than their counterparts in Cluster 3 (PSS: 3: 16.6 ±5, p = 0.02 BDNF: 10828 ± 1127 pg/mL, p = 0.08). Men in Cluster 1 differed in terms of stress (19.8 ± 7 vs. 21 ± 7.5 score) and BDNF levels (Cluster 1: 5204 ± 818 vs. Cluster 2: 7656 ± 843 pg/ml, p = 0.002) but not in the number of years living with HIV. The proportion of subjects with multiple mood comorbidities was disturbingly higher (26%), and all were members of Cluster 1. Multiple logistic regression analyses indicated that participants reporting high relative to low levels of perceived stress, dual mood comorbidity, altered BDNF levels and low income increased the likelihood of being a member of Cluster 1.

**Conclusion:**

This study found that stress and overlaying psychiatric comorbidities are linked with persistent alcohol use. Findings suggest that BDNF and social support seems to be a logical target as it seems to be the bridge linking mood disorders and alcohol consumption.

## Introduction

While in the beginning of the HIV epidemic concerns were primarily related to drug addiction. Through the years hazardous alcohol use has occupied a prominent place in the HIV/AIDS epidemic [1–6]. Although HAU is a topic of theoretical interest for both, researchers and health care providers, given its excessive rates (40–80%) [2–6], little information is available with regards to the underpinnings mediating the excessive rates among PLWH. Such information can help in the development of health policy tools, but it can also guide the design of successful treatment approaches, which, so far, have produced only limited evidence that such interventions work among PLWH [7].

One mechanism that could explain the excessive rates of HAU among PLWH is Brain Derived Neurotrophic Factor (BDNF), a “Miracle Grow” chemical for the brain that could be directly affected by the HIV virus, as well as by epigenetic factors. Unequivocal experimental and clinical evidences, causally linked alterations of BDNF signaling with the pathophysiology of alcohol abuse [8–11]. In animal models, BDNF depletions provoked anxiety-like behaviors, resulting in increased alcohol intakes, which could be rescued by BDNF co-infusion [11]. BDNF heterozygous mice, which expressed about half as much BDNF protein as their wild-type counterparts, displayed increased conditioned place preferences and locomotor sensitization to alcohol [10–13]. They also showed prolonged alcohol consumption, following a period of abstinence, suggesting that BDNF may decrease the rewarding effects of alcohol [13]. In fact, BDNF has been suggested as a predictor of relapse. The region of human chromosome 11 containing BDNF has been implicated as a susceptibility locus for severe alcohol withdrawal [14]. Activity-dependent activation of BDNF has been linked with the neuroadaptation process that occurs in the development of alcohol addiction [10,15–17].

**Figure 1:**
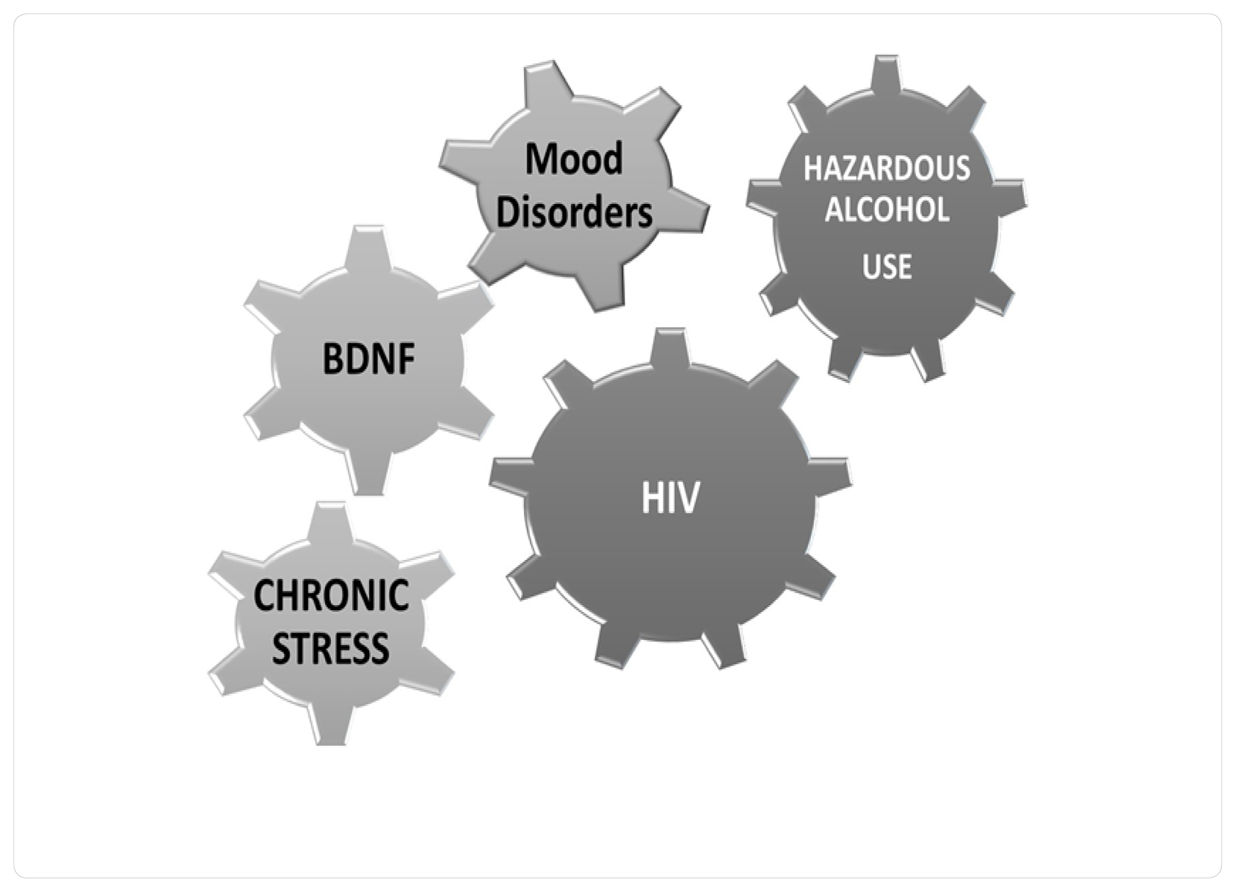
A proposed model of the interplay between Mood Alcohol and BDNF status among people living with HIV.

As depicted in Figure 1, studies have demonstrated a reciprocal relationship between mood disorders and BDNF levels, both in circulation as well as in the brain [18]. Both, acute and chronic stress may alter BDNF levels [19,20]. Acute stress caused by immobilization, as well as swim-stress tests, increased the levels of BDNF mRNA, suggesting that epigenetic mechanisms underlined this response [19,20]. Increased BDNF expression may represent a protective mechanism in response to stress. Conversely, reduced BDNF levels after exposure to repetitive and chronic stress has been observed and appears to represent an adjustment of this mechanism [21–23]. The relationship between stressful life events and hazardous alcohol use is also recognized in humans. Epidemiological studies have consistently demonstrated that a variety of stressors lead to drinking. PLWH are being exposed to a myriad of societal (poverty) and HIV related stressors (stigma). However, the relationship between BDNF, stress, and alcohol use has not been assessed among PLWH. Such information is of high relevance for the design of future interventions.

The World Health Organization foresees depression as becoming the leading cause of worldwide disability [24]. Nearly one-third of people with major depression also have an alcohol problem, according to one major study conducted by the National Institute on Alcohol Abuse and Alcoholism [25]. Of concern, depression is a widespread problem among PLWH. Heckman et al. found that among PLWH, 29% had moderate to severe depression, with an additional 31% having mild depression [26]. As many as half of participants enrolled in the Gay Men’s Health Crisis and the America’s Research on Older Adults with HIV, had depression. In many cases depression may be the first one to occur, and in others it emerged after HIV diagnosis [27]. Unfortunately, people struggling with depression and HIV are also likely to struggle with alcohol abuse and vice versa [28]. It seems that by altering serotonin, BNDF is the bridge that connects alcohol and depression.

On the same track, Pandey and colleagues have demonstrated that BDNF deficiency induced anxiety in rats, followed by increased consumption of alcohol [12]. By blocking BDNF levels in the amygdala, the researchers were able to trigger both anxiety like behaviors and alcohol dependence. Noteworthy, this area of the brain deals with stressful events, as well as with rewarding experiences of drugs and alcohol. Recently, You Chang demonstrated that high alcohol-drinking rats had innately higher anxiety levels, along with less BDNF expression [29]. When levels of BDNF in the central and medial amygdala were restored to normal, anxiety and alcohol consumption diminished, suggesting that the deficits in BDNF signaling were the key in these disorders. In humans, anxiety is frequently measured using the State Trait Anxiety inventory, a test that emerged from the distinction between anxiety as a mood state that is temporary, and anxiety as a stable attribute. Studies suggest that individuals with trait anxiety use alcohol as a mechanism to cope with the symptoms. According to these postulates, trait anxiety levels are positively associated with alcohol dependence [30–32].

Another mood disorder that is highly prevalent among PLWH, and that has been associated with alcohol use, is stress [28,33,34]. In terms of health, any event that exerts a physical, emotional, or cognitive demand on the individual is considered a stressor. Yet, when it occurs in quantities greater than the individual’s capacity to handle, pathological changes can occur [34]. Stress is considered a significant factor in the initiation and continuation of HAU [35]. Researchers have postulated that the body’s response to stress most likely plays a role in the vulnerability of the initiation of HAU, and in relapse in those under treatment [33–35]. This relationship probably is mediated, at least in part, by common neurochemical systems, such as the serotonin, dopamine, and opiate peptide systems, as well as the Hypothalamic-Pituitary-Adrenal (HPA) axis [36–38]. The basic mechanisms are still under investigation, but involve release of the stress hormones (i.e. corticosterone), causing alterations in BDNF, the serotonin pathways, and neurogenesis. Further in the study, these animals showed increased alcohol self-administration, regardless of whether the stressful experiences continued during the length of the study or had ended weeks earlier [39]. Remarkably, both acute and chronic stress resulted in an aberrant regulation of BDNF signaling in the hippocampus, prefrontal cortex, and amygdala [21–23]. The initial response is a compensatory elevation of BDNF to prevent damage, which is followed by a drop in BDNF levels. BDNF deficits may lead to an increased preference for ethanol.

In summary, it has been clearly established in animal models that anxiety, depression, and stress, could trigger alcohol use; however, to the best of our knowledge, these relationships have not been explored among PLWH. Examining their specific relationships with the excessive rates of alcohol use in this population is of critical importance. Accordingly, the dual goal of this research is: 1) to investigate the often assumed relation between alcohol use and mood disorders, and 2) to determine if similar conclusions can be derived in humans. In addition, to examine HAU at the neurochemical level (BDNF) in a cohort study of PLWH receiving care in South Florida, a region that has been disproportionately affected by the HIV epidemic. Our long-term goal is to inform clinical intervention efforts aiming to reduce the deleterious effects of HAU in PLWH.

## Methods

### Study population

Data was collected in South Florida, as part of the Platelets Mediating Alcohol and HIV Damage Study (PADS). All subjects signed both, written informed consent and HIPAA forms. The study consists of 400 PLWH, who were at least 18 years old and under regular care at primary open-access public health systems in Miami. Subjects with major comorbidities were not eligible: CNS opportunistic infection, head injury with or without loss of consciousness, tumors, major psychiatric disease, developmental disorders, severe malnutrition, or confirmed cardiovascular or immune based disease, (i.e., malignancies, autoimmune diseases, arthritis). To reduce the confounding effects of liver disease and/or illegal drug use, we also excluded injection and dependent drug users.

### Alcohol drinking

At each visit, using a non-judgmental interview style, participants was asked about alcohol intake in the past six months, using the Alcohol Use Disorders Identification Test (AUDIT) and the Alcohol Dependence Scale (ADS) [40–42]. Participants were asked to report a serving size using models of 12 ounces of beer, 5 ounces of wine, or 1.5 ounces of liquor. Alcohol consumption scores were computed by averaging cross products of quantity and frequency of beer/wine and hard liquor. Then, based on the National Institute of Alcohol Abuse and Alcoholism criteria, the sample was dichotomized in two groups: hazardous (men who reported >14 drinks/week or >4 drinks in one day, and women who reported >7 drinks/week or >3 drinks in one day) or non-hazardous alcohol users, for those who reported fewer drinks. Participants who drank more than five standard drinks in a given day were considered binge drinkers [43].

### Participants’ assessment protocol

Those participants who expressed a willingness to participate and that provided written informed consent, as well as a medical release, were consecutively enrolled and followed for a period of 6 months. Research staff who were trained in social and medical sciences fields, and supervised by the research coordinator and the licensed clinical psychologists, collected information using structured questionnaires.

### Depression

The BDI-II was completed by a trained assessor, and results were further checked by a professional, to ensure quality control. The BDI is one of the most widely used instruments to screen and to measure the intensity of depression, and has been translated into several languages [44]. The BDI contains 21 items that can be answered on a 4-point scale. The total score, which ranges from 0–63, is obtained from the sum of the scores of the 21 items. A total score of 0–9 is considered to be normal, 10–18 corresponds to mildly depressed, 19–28 equals moderate depression, and scores above 29 are considered severe depression.

### State-trait anxiety inventory form X

Trait anxiety was measured with Spielberger’s State-Trait Anxiety questionnaire (STAI) [45,46]. STAI is composed of two scales for assessing levels of state and trait anxiety. For the 20-item state anxiety scale participants have to rate each question on a 4-point Likert scale (from “almost never” to “almost always”). Scores of 20–39 were indicative of low anxiety; 40–59 moderate, and from 60–80 severe anxiety.

### Perceived stress scale

Developed by Cohen and colleagues, this instrument is a global measurement of stress. Participants responded to 10 questions, to assess whether they perceive their lives as unpredictable, uncontrollable or overloaded [47,48]. The PSS is a widely used scale to measure stress in chronic conditions, and therefore we deemed appropriate to be used in PLWH. The PSS is not a diagnostic test, and therefore there are no cut-offs to determine stressed individuals. Accordingly, stress levels were analyzed by quartiles.

### Brain derived neurotrophic factor (BDNF)

Prior studies have demonstrated that plasma levels of BDNF, although different from levels in Cerebrospinal Fluid (CSF), are correlated with CSF in Central Nervous System (CNS) diseases [49,50]. Due to this association, circulating levels of BDNF were selected for analyses. Plasma BDNF levels were measured using a commercially available ELISA kit (R&D System), according to the manufacturer’s instructions. Briefly, 50 μl of standard and 20-fold diluted samples were pipetted into wells of a 96-well immunoplates. An enzyme-linked monoclonal antibody specific for BDNF was added to the wells. Following a wash to remove any unbound antibody-enzyme reagent, a substrate solution was added to the wells, and color developed in proportion to the amount of BDNF bound in the initial step. The color development was stopped, and the intensity of the color was measured. BDNF concentration in plasma was calculated based on a standard curve. The minimum detectable dose of BDNF is typically less than 60 pg/mL.

### Potential confounders

A number of plausible confounders were measured, and included age, sex, race/ethnicity, years of education, and smoking status. Overall, nutritional status was determined by measurements of serum albumin levels. In addition, HIV associated variables were assessed, as time since diagnosis, time receiving antiretroviral therapy, HIV-specific clinical history, CD4 and Viral load (AMPLICOR HIV-1 monitor test, Roche Diagnostics, Branchburg, NJ).

### Statistical analyses

The normality of the distribution of primary outcomes of interest was examined with a normal probability plot. Descriptive statistics, such as minimum, maximum, median, and mean with SD were used to summarize the data, as well as to detect outliers and missing values. Group comparisons were assessed using the chi-square test for categorical variables, two sample Student’s t-test for normally distributed continuous variables, and the Wilcoxon rank sum test for non-parametrically distributed continuous variables. We employed the Bonferroni correction, because of the multiple comparisons. Alcohol use variables, BDNF, BDI, STAI and PSS were assessed both as continuous as well as categorical variables.

Level of alcohol use has been the most frequently modeled dependent variable in the extant literature on alcohol, and was selected to analyze drinking trajectories. To examine potential mediators, regression models were employed, using the interaction terms as independent variables and BDI and Anxiety, and PS scores as dependent variables. All plausible predictors used on multivariable regression models were dichotomized, to give equal weight to the different variables associations. Psychological measures were dichotomized in accordance to values for distress (PSS >23), mild depression (Beck > 20) and anxiety (state> 35, and trait anxiety > 35) years living with HIV by 5 years, CD4 counts < 200 yes/no, viral load detectable yes/no, therapy yes/no, smoking yes/no, sociodemographics and therapy for mood disorders yes/no. Analyses were controlled by age, cigarette smoking, HIV, BMI, time of HIV diagnosis and treatment. The validity of model assumptions was evaluated using analysis of residuals. P values less than 0.05 (2-tailed) were considered significant. Data analyses were performed using SPSS version 18.

### Ethics

The study was approved by Florida International University and the University Of Miami School Of Medicine Committee for the protection of the rights of human subjects.

## Results

### Group characteristics

The 400 participants included 37% females and 63% males, ranging in age from 21 to 50 years (42 ± 6 years). Participants had received a diagnosis of HIV infection typically more than a decade before (15 ± 8 years ago range 2–29).

Though among non-HAU over half (55%) never or rarely drank alcoholic beverages (Control Group), 34% imbibed at least once a week, typically more than two drinks per occasion. HAULWH were actively drinking at the time of assessment an excess of 7 ± 5 drinks per day. They regularly consumed alcohol on average 4 days a week, and the mean total intake was of 31 drinks/week.

Age, race/ethnicity, sexual orientation for men, education level, and health insurance status were not associated with being or not a HAU.

### Depression and alcohol use

The average BDI total score was 17.3 ± 12.4 in the “mild depressed” range. Of concern, a sizable portion of the group met the criteria for mild (16%), moderate depression (6%) or severe depression (6%, with scores of 30 and above). As depicted in Table 1, depression was not associated with either socio-economic status or education. Although depression is the leading cause of disease burden for women, in our sample differences in the rates of depression by gender were not significant (17.7 ± 15.6 ± 12 score, p = 0.1). Importantly, depressed participants were more likely to report heavy drinking behavior (OR=2.5; p = 0.006).

### BDNF and depression

There was a wide concentration range of BDNF in circulation from 298 to >20,000 (mean 8384 ± 6366 pg/ml). BDNF levels differed by gender with women exhibiting the highest levels (9958.9 ± 6578 vs. 7470 ± 6068 pg/ml, p = 0.0001). Our data showed that participants with BDNF <3,000, were more likely to score in the moderate to severe range of the BDI-II (OR=1.7: 95% CI 1–2.99, p = 0.05).

### Anxiety and alcohol drinking

Anxiety disorders were also prominent among the sample, with 90% of the PLWH affected. The mean score of the group was 42.33 ± 14. S-anxiety scores between 28–50 were reported in 46% of the subjects, and 36% had scores above 60. Anxiety scores were similar between males and females (39.0 ± 15 vs. 38.7 ± 14, p = 0.8). However, low income individuals were 5 times more likely to have anxiety scores above 35 than the more economic solvent counterparts (OR=4.8 95% CI 2–11.4, p = 0.0001)

Since among the many reasons why anxiety could be developed were the deteriorating symptoms of HIV disease, we examined this association. However, neither CD4 nor viral load varied between the groups.

At baseline, a positive correlation between alcohol use and higher trait anxiety was observed (r = 0.1, p = 0.04). For additional analyses participants were dichotomized above and below 35. Subjects with low or no anxiety drunk on average one day more per week (3 ± 2.5 vs. 2.1 ± 2 days, p = 0. 004) and overall more drinks per week (16 ± 1 vs. 12 ± 1 drinks, p = 0.04).

### Perceived stress and alcohol drinking

Mean PS score was 18 (SD = 6.7, median = 20.0, extremes = 15–23) in the 400 participants. PS scores did not differ according to gender (18.7 ± 6.8 18.1 ± 6.7, p = 0.5), CD4 (< 200 counts) or viral load. However, individuals in the lowest income quartile reported significantly higher PS scores than the rest of the sample (18.3 ± 6 vs. 15.5± 6.5, p = 0.04).

Analyses indicated that subjects with high stress levels (top quartile) did not report an earlier use of alcohol. The increase of perceived stress coincided with a significant increase in the mean levels of drinking per week. As depicted in Table 2, high levels of perceived stress were associated with an increase in the frequency of alcohol use (3 vs. 5 drinking days, p = 0.02), as well as in the number of drinks taking per occasion. These findings indicate that greater stress levels were associated with the drinking profile in a dose dependent manner. Notably, a significant correlation between PSS and AUDIT was also observed (r = 0.247, p = 0.0001).

Since in animal models a decreased Brain-Derived Neurotrophic Factor (BDNF) expression and/or function, particularly in hippocampus, have been implicated in the pathophysiology of stress-related disorders, we explored such a relationship. While total PSS were not significantly associated with BDNF levels, additional analyses indicated a negative correlation between BDNF levels and reporting feeling nervous and stressed (r=−0.192; p = 0.012), in which lower BDNF levels were associated with the PSS. Stratification of data on the basis of feeling stress fairly often (scores 3 and 4) revealed a positive correlation between low BDNF levels and feeling distressed (scores 3 and 4: 3951 ± 546 vs. scores 0–2: 5954 ± 668 pg/mL, p = 0.02). Notably, feeling nervous and stressed were also co-related with AUDIT scores (r= −0.197, p = 0.003).

### Longitudinal analyses

A total of 320 out of 400 subjects completed the follow-up assessment, and have completed data for longitudinal analyses. A significant change in depression was noted at the 6-month evaluation, as the mean BDI scores decreased by 19% (− 3.0 ± 0.8, p = 0.000). Evaluation of STAI, on the contrary, revealed significant increases (2.6 ± 1.1, p = 0.02). Stress scores were similar in the two evaluations (−0.7 ± 0.5, p = 0.1).

Even though at first glance the overall group decreased their alcohol intakes from baseline to the follow-up visit (−5.9 ± 1 drinks per week), a cluster analyses shows three distinctive trajectories. The first one, revealed a group with increased drinking (Cluster 1: n=140), constant alcohol intake (Cluster 2: n = 60), and one with decreased consumption (Cluster 3: n =120).

These clusters are consistent with prior studies describing 3–4 types of alcohol users: a nonuser/low-use class, a chronic or high-use class, and a desisting class. Since BDNF and drinking phenotypes differed by gender, we constructed separate models for male and females. As depicted in table 3, women in Cluster 1 reported less years living with HIV than the other two groups. Women in Clusters 1 and 2 have lower BDNF levels than their counterpart in Cluster 3. Women in Cluster 1 and 2 also exhibited higher levels of stress. Among men Group 1 differed in terms of stress and BDNF levels, but not in the number of years living with HIV. Regarding age, education and income, among women there were no significant differences among the 3 classes.

In the 3 trajectory group solution for men, Cluster 1 (both men and women) showed moderate increasing in drinking (from 4 to 6.2 drinks per occasion= +2.3 ± 0.6, p = 0.001); Cluster 2 showed low desisting in drinking (from 1.2 to 1 drink per occasion p = 0.4). Class 3 significant desisting, while reported initial drinking at a similar or even higher level than Cluster 1 (6.9 ± 4 drinks per occasion), informed only 2.8 ± 0.2 drinks in a row, at the follow-up visit (p = 0.0001).

Surprisingly, analyses uncovered higher AUDIT scores (both men and women) across the clusters with Cluster 1 being followed by Clusters 2 and 3 (1: 14.5 ± 8 vs. 2= 6.6 ± 4.2, vs. 3=8.7 ± 7.5, p = 0.001). In addition, dual diagnoses (anxiety + stress, or depression +stress), which were present in 26% of the study population may complicate alcohol use trajectories as analyses indicated that they were all members of Cluster 1.

Table 4 summarizes regression results for alcohol drinking at the last visit. Multiple mood comorbidities and distress were uniquely associated with increasing alcohol drinking during the length of the study. Data indicated that mood and BDNF were independent predictors of alcohol use disorders.

## Discussion

Several significant findings have emerged from this study. Of concern, our data indicated an astonishing prevalence of mood disorders in our study population. In our cohort, as many as 38% fulfill the criteria for depression and 63% for anxiety (scores >35). This is significantly higher than those reported by Pence and colleagues [51], in which 20% had an anxiety disorder. Of most concern is the sizable proportion of the samples that have dual or triple mood comorbidity (stress, anxiety, depression). However, high rates were not unexpected, given that our sample was predominantly formed by minority patients (women, Hispanic and African Americans), with lower-than-average incomes. As such, these individuals represent an especially vulnerable population. Although, acute and transient stress is not harmful, when stress becomes chronic, the body does not return to the basal unstressed level. This chronic state while more subtle continues to exert effects on the autonomic system leading in the long-run to metabolic abnormalities, insulin resistance, and endothelial damage [33–38]. Notably, our data provide novel evidence for the unique effects of low BDNF as a prospective risk factor for hazardous alcohol use among PLWH. The cognition may also be compromised maintaining this vicious cycle.

It is surprising that the relationship between mood disorders and alcohol use has not received more empirical scrutiny. The analyses showed that high levels of perceived stress and anxiety, but not depression, are associated with high levels of alcohol use. Self-reported symptoms of apathy are quite common among HIV-infected individuals. Given the enduring prevalence and clinical significance of negative moods among PLWH, examination of malleable processes related to them is relevant from an intervention standpoint.

It is concerning that almost a third of the participants have multiple comorbidities (stress + anxiety + depression), revealing that these individuals were in need of treatment for their psychiatric symptoms. These rates are far higher than those reported in the National Comorbidity Survey Replication of 9% of the adult population. These concomitant problems seriously threaten health, not only because it negatively impacts clinical outcomes, but also adherence to HIV medications [51]. In addition to physical health and well-being, HIV and aging can have profound effects on the brain, making aging PLWH more susceptible to negative mental health outcomes such as depression, and anxiety. Our findings indicating that these individuals with dual or triple comorbidity are more likely to be hazardous alcohol users and suggest that the underlying mechanisms of these disorders are the same or are highly overlapping.

In this article, we have uncovered one common mechanism mediating maintenance of hazardous alcohol use, and stress: BDNF. Our observation is consistent with recent studies demonstrating that the brain stress systems, particularly the CRF pathway, play an important role in the maintenance of HAU. Studies have shown that chronic alcohol exposure alters the density of dendritic spines in the amygdala [33,52,53]. BDNF-Arc signaling pathway has been postulated to mediate the spine density changes that heightened greater alcohol intake [52]. BDNF may be particularly important, as it is a key regulator of both dopamine and serotonin pathways, which regulates both drinking behaviors as well as responses to emotionally salient triggers. These findings are highly relevant for PLWH, given that negative moods and alcohol use disorders play a prominent role in the clinical presentation of the disease, and may cause a disruptive impact on the quality of life of the affected subjects.

Our study also highlights the importance of gender analyses given different drinking, BDNF and even HIV profiles. For example, despite similar age, women have been living longer with HIV, they have a shorter story of drinking, yet their drinking profiles are getting closer to those of males. Gender differences in alcohol consumption were more notable for quantity rather than frequency. Since studies have indicated that older individuals tended to have lower BDNF levels it will be important to closely follow-up with these individuals as increased aging of the brain will aggravated these problems [54–56].

Nevertheless, our results have some limitations: First, a short time follow-up has reduced our capacity to perform more detail analyses. Second, the study design did not allow us to generalize study findings as our population was limited to those living in South Florida. However, the sample reflects the epidemiology of those around the United States. Despite these limitations the high prevalence of mood comorbidity and HAU in this population underscores the importance of integrating mental health screenings and treatments into clinics that provide services for PLWH. These results indicate that health care providers must be attentive for signs of hazardous alcohol use among subjects with multiple mood disorders. Our findings emphasize the need for additional research focused on BDNF. Notably, antidepressants including serotonin re-uptake medications did not demonstrate any effect, indicating that other types of pharmacological interventions need to be developed and tested.

## Figures and Tables

**Table 1:** Sample Characteristics.

Variable	HAU N=200	Non-HAU N=200	P value
Age (Years)	43 ± 6.4	41 ± 6	0.7
**Gender** Men	67%	60%	0.1
Women	33%	40%	
**Race/Ethnicity** African American	70%	66%	0.4
Black Caribbean	2%	4%	
Hispanic	22%	23%	
White	6%	7%	
**Annual Income:** Less than $10,000	88%	86%	0.7
$11,000–$20,000	8%	10%	
$20,000–$49,000	2%	2%	
>$50,000	2%	2%	
**Education** (years of school)	11.5 ± 2	11.3 ± 2.4	0.1
**Albumin** mg/dl	4 ± 0.4	4.1 ± 0.5	0.9
**CD4 cell Counts**	408 ± 259	455 ± 310	0.1
**Viral Load** Log	2.7 ± 1.3	2.6 ± 1.3	0.4

**Note:** Demographic characteristics were expressed as percentages by HAU groups. Biological measures were presented as means and standard deviations

**Table 2:** Stress and Alcohol Profile.

Variables	Non Stressed 1^st^ quartile	Mild Stressed 2^nd^ Quartile	Severe Stressed 3^th^ Quartile	P value
**Years living with HIV**	16.6 ± 7.2	15.3 ± 7.7	14.1 ± 7.5	0.04
**Years Drinking**	6.0 ± 1.3	8.1 ± 1.2	7.2 ± 1	0.4
**Past Drinking/week**	8.3 ± 1.9	11.3±1.7	12.9 ± 1.6	0.07
**Baseline Total Drinking/week**	11.4 ± 2.6	18.4 ± 3.3	19.9 ± 2.5	0.01
**Number of Drinks/day**	2.9 ± 0.4	4.2 ± 0.5	5.1 ± 0.5	0.002
**Number of Days per Week Drinking**	2.2 ± 0.3	2.8 ± 0.3	3.3 ± 0.3	0.02

Values are means ± SD or percentages

**Table 3:** Characteristics of the PLWH According to Gender and Alcohol Clusters.

	Men n = 250				Women n = 150			
Variables	Group 1	Group 2	Group 3	P value	Group 1	Group 2	Group 3	P value
**Age (years)**	42 ± 6	43 ± 6	42 ± 6	0.3	44 ± 4	44 ± 5	43 ± 6	0.8
**Years with HIV**	14 ± 8	15 ± 7	14 ± 8	0.6	12.5 ± 8	18 ± 6	17 ± 7	0.06 0.03
**Years Drinking**	9 ± 1.1	10 ± 1.5	8 ± 1.7	0.5	7 ± 3.2	11 ± 1.9	4 ± 0.9	0.1
**Education**	11 ± 2.3	12 ± 2	11 ± 2.2	0.2	11 ± 2.3	11 ± 2.4	11 ± 1.9	0.7
**Anxiety**	39.2 ± 14	40.1 ± 14	39.4 ± 16	0.7	38.4 ± 14	38.6 ± 12	39.3 ± 15	0.8
**BDI**	18.2 ± 12	16.3 ± 10	16.5 ± 11	0.3	17.7 ± 9	14.0 ± 11	16.3 ± 12	0.3
**Perceived Stress**	19.8 ± 7	17.6 ± 6	14.3 ± 8	0.08	21.0 ± 7.5	19.3 ± 7	16.6 ± 5	0.02
**BDNF pg/ml**	5204 ± 818	7656 ± 843	8523 ± 648	0.002	7904 ± 124	10405 ± 909	10828 ± 1127	0.08

**Table 4:** Multivariate Analyses.

Parameter	B	95% Wald Confidence Interval			
		Lower	Upper	Wald Chi-Square	Sig.
**(Intercept)**	−0.410	−1.220	0.400	0.985	0.321
**Comorbidities**	0.035	0.019	0.050	19.073	0.000
**Stress quartiles**	0.280	0.027	0.533	4.692	0.030
**BDNF**	3.379E-5	1.397E-5	5.360E-5	11.171	0.001
**Income**	0.313	0.004	0.622	3.942	0.047
**Number of Children**	0.153	0.048	0.257	8.195	0.004
